# Short structured feedback training is equivalent to a mechanical feedback device in two-rescuer BLS: a randomised simulation study

**DOI:** 10.1186/s13049-016-0265-9

**Published:** 2016-05-13

**Authors:** Noemi Pavo, Georg Goliasch, Franz Josef Nierscher, Dominik Stumpf, Moritz Haugk, Jan Breckwoldt, Kurt Ruetzler, Robert Greif, Henrik Fischer

**Affiliations:** Department of Cardiology, Medical University of Vienna, Vienna, Austria; Department of Anaesthesia, General Intensive Care and Pain Control, AUVA Lorenz Böhler Trauma Hospital, Vienna, Austria; Hospital of the Sisters of Charity Linz, Linz, Austria; Department of Emergency Medicine, Medical University of Vienna, Vienna, Austria; Faculty of Medicine, University of Zurich, Zurich, Switzerland; Institute of Anaesthesiology, University and University Hospital Zurich, Zurich, Switzerland; Department of Anaesthesiology and Pain Therapy, University Hospital Bern and University of Bern, Inselspital, 3010 Bern, Switzerland; Federal Ministry of the Interior and Sigmund Freud University Vienna, Vienna, Austria

**Keywords:** Cardiopulmonary resuscitation (CPR), Basic life support (BLS), Feedback device

## Abstract

**Background:**

Resuscitation guidelines encourage the use of cardiopulmonary resuscitation (CPR) feedback devices implying better outcomes after sudden cardiac arrest. Whether effective continuous feedback could also be given verbally by a second rescuer (“human feedback”) has not been investigated yet. We, therefore, compared the effect of human feedback to a CPR feedback device.

**Methods:**

In an open, prospective, randomised, controlled trial, we compared CPR performance of three groups of medical students in a two-rescuer scenario. Group “sCPR” was taught standard BLS without continuous feedback, serving as control. Group “mfCPR” was taught BLS with mechanical audio-visual feedback (HeartStart MRx with Q-CPR-Technology™). Group “hfCPR” was taught standard BLS with human feedback. Afterwards, 326 medical students performed two-rescuer BLS on a manikin for 8 min. CPR quality parameters, such as “effective compression ratio” (ECR: compressions with correct hand position, depth and complete decompression multiplied by flow-time fraction), and other compression, ventilation and time-related parameters were assessed for all groups.

**Results:**

ECR was comparable between the hfCPR and the mfCPR group (0.33 vs. 0.35, *p* = 0.435). The hfCPR group needed less time until starting chest compressions (2 vs. 8 s, *p* < 0.001) and showed fewer incorrect decompressions (26 vs. 33 %, *p* = 0.044). On the other hand, absolute hands-off time was higher in the hfCPR group (67 vs. 60 s, *p* = 0.021).

**Conclusions:**

The quality of CPR with human feedback or by using a mechanical audio-visual feedback device was similar. Further studies should investigate whether extended human feedback training could further increase CPR quality at comparable costs for training.

## Background

Immediate cardiopulmonary resuscitation (CPR) is considered the most important life-saving intervention for sudden cardiac arrest (SCA) [1, 2]. Reported outcomes of SCA vary largely between different emergency systems, regions and facilities [3–5]. Despite many studies having demonstrated the importance of high quality CPR on patient outcome [6–10], most CPR does not meet these criteria [10] and even healthcare practitioners exhibit sub-optimal performance [11]. Experts agree that without understanding CPR performance, an improvement of performance, which could prevent many deaths due to cardiac arrest, cannot occur [4].

To address this issue, the 2010 International Liaison Committee on Resuscitation (ILCOR) guidelines highlighted the importance of optimising the resuscitation process and CPR parameters while endorsing the use of feedback devices in clinical practice as part of a comprehensive system of care for cardiac arrest [8]. Good evidence supports the use of feedback devices during training to achieve CPR parameters closer to guideline criteria [12], and to improve skill acquisition [13, 14] and retention [15]. A systematic meta-analysis of feedback devices in human and manikin studies simulating CPR scenarios showed chest compression parameters closer to recommendations, however there is no evidence that this would translate into better patient outcomes [12].

While knowledge about CPR quality is constantly growing, its optimal application in clinical practice remains challenging. Feedback devices could help us improve CPR skills and CPR quality. However, it is not known whether alternative methods to supply feedback might be as effective. There could also be limitations of mechanical devices, since feedback is restricted to the parameters measured. In addition, mechanical devices are not ubiquitous, neither for training, nor for real CPR.

The aim of our study was to investigate in a two-rescuer scenario whether feedback from trained humans could be as effective for CPR quality as from a mechanical audio-visual feedback device. We hypothesised that continuous feedback during CPR improves CPR quality irrespective of feedback type.

## Methods

### Study participants

We enrolled undergraduate third year medical students in this open, prospective, randomised, controlled parallel group study. The study was performed at the Medical University of Vienna, a university-affiliated tertiary care centre, over a period of 4 weeks in October and November 2013. Students with basic BLS skills were recruited from the second unit of a compulsory BLS training course of the curriculum. Upon inclusion in the study, all students had received 2 h of basic BLS training on a manikin following a standardised teaching protocol of the Medical University of Vienna according to the ILCOR guidelines for adult automated external defibrillator BLS (AED-BLS) [8]. Study participants had to be 18 years or older and provide written informed consent. Study participation was voluntary. Students unable to perform BLS (e.g. physical ability due to injury) were not eligible. The study protocol was in line with the Declaration of Helsinki and was approved by the local Ethics Committee of The Medical University of Vienna (EK No. 1754/2013). Informed consent was obtained from all participants and they were assured that participation would not influence their grades for the course.

After providing written informed consent, students were randomised in 3 groups using block randomisation by means of sealed envelopes. The envelopes were generated by the Department of Statistics, assuring a random allocation sequence with equal allocation ratios. After assignment to the group, students were put into pairs according to the assignment of randomised codes. Prior to study measurements, all participants received initial training using a modified 4-stage approach [16, 17]: following a standardised video according to their group, students were allowed to practice their basic life support (BLS) scenario under supervision until they felt sufficiently confident with the method. The duration of the training videos and the free practice differed between the different groups. After completion of training, participants performed 8 min of two-rescuer BLS with bag-valve mask ventilation according to ERC 2015 guidelines [18].

### Study groups

- The standard BLS (sCPR) group performed resuscitation with a compression to ventilation ratio of 30:2 with a change of rescuer position after 2 min as recommended by the ERC 2010 guidelines [8].
- Students in the human feedback (hfCPR) group were trained to specifically consider five pre-defined CPR parameters, i.e. compression rate, compression depth, correct hand position, correct decompression and hands-off time. The study participant performing ventilation was instructed to give verbal feedback about the aforementioned CPR parameters to the rescuer performing chest compressions at the beginning of each new cycle and to correct in any case of deviation from the trained criteria. The teaching video highlighted these five parameters showing examples of correct CPR and deviations in each of the parameters one by one with verbal corrections from the ventilating participant.
- The QCPR feedback device (mfCPR) group performed BLS using the HeartStart MRx with Q-CPR-Technology™ (Philips, Netherlands) feedback device. The device is connected to the MRx™ defibrillator and placed over the sternum of the manikin right under the hands of the rescuer. An integrated accelerometer and a pressure sensor measure compression rate and depth. Real time visual feedback is provided using graphs and numbers on the display of the MRx™ defibrillator placed next to the manikin. Furthermore, automated audio feedback advises the rescuer about necessary corrections if values diverge from the programmed range.

CPR quality was assessed by using two Ambu®ManC manikins (Ambu, Ballerup, Denmark) with medium thorax resistance. The manikins were placed on a firm even floor to avoid inaccuracies in measuring compression depth and were connected to personal computers (Fujitsu Siemens, Amilo PA 1510). Data recording and analysis were performed with the Ambu® CPR Software (version 2.3.9, Ambu®, Ballerup, Denmark) as previously described [19]. Prior to the start of the study, the accuracy of sensing compression depth was assessed and set to various compression depths between 10 and 63 mm using Thumper® model 1005 (Michigan Instruments Inc., Grand Rapids, MI) [19]. Bag-valve mask ventilation (Laerdal LSR Adult Standard, Stavanger, Norway) was performed at a compression-to-ventilation ratio of 30:2.

### Measurement and outcomes

Effective compression ratio (ECR), a parameter combining correct hand position, chest compression depth, and complete decompression multiplied by flow-time fraction, which has recently been established to assess quality of CPR, was defined as the primary outcome parameter [16]. The primary hypothesis to be tested was that there is no difference in ECR between the groups sCPR, hfCPR and mfCPR. In brief, ECR was defined as effective compressions in % (EC: correct hand position, depth (50–60 mm) and complete decompression) multiplied by flow-time fraction (FTF) in %. Complete decompression was considered sufficient with no residual leaning of more than 10 mm in accordance with previous studies to eliminate artefacts [20]. The ratio of ECs during CPR duration was assessed by a Visual Basic based excel macro and then multiplied by the FTF. With a 30:2 compression-to-ventilation ratio and a resulting FTF of 79 %, a guideline compliant ECR would be indicated by 0.79.

Secondary outcome parameters of chest compressions such as EC, compression rate (CR), compression depth, complete decompressions and incorrect pressure point were assessed. Additionally, time related parameters such as FTF (flow time fraction of chest compressions), absolute hands-off time (time fraction without compressions or ventilation) and time until first chest compression as well as ventilation parameters such as ventilation volume, ventilation minute volume, ventilation time and the number of gastric inflations were evaluated.

### Subjective assessment by study participants

Following the BLS testing, all study participants were requested to complete a form focusing on their experience with the applied method. Answers were rated on a 10-point Likert scale (most difficult = 1 to easiest = 10). As a final question, participants were asked which method they would prefer in a real-life BLS situation.

### Statistical analysis

The CRP related primary and secondary outcome parameters were regarded pairwise for the 8-min CPR period, while baseline characteristics of the participants and the subjective assessment forms were analysed for each study participant separately. Based on previous studies [19], we estimated a standard deviation of 0.21 for our primary study endpoint (effective compression ratio). Hence, based on a two-sided *t*-test with an alpha = 0.0167 (Bonferroni corrected for all pairwise comparisons between the 3 groups) and a power of 80 %, a total sample size of 60 study participants per group was needed. To adjust for potential dropouts, we estimated a dropout rate of 10 % and therefore increased the group size to 70 participants per group. Sample size calculation was performed using NQuery 6.01 (Statistical Solutions Ltd., USA). Normal distribution was tested by visual assessment of the investigated variables. Normally distributed continuous data was presented as mean ± standard deviation (SD) and compared using Student’s *t*-test. In the absence of normal distribution, continuous variables were expressed as median and interquartile range (IQR) and compared using Kruskal-Wallis statistics. Analysis of variance (ANOVA) was used to compare all three groups. Frequencies were compared by means of the Chi-Square test. SPSS 21 for Mac (IBM SPSS, USA) was used for all analysis. *P*-values <0.05 were considered statistically significant.

## Results

**Fig. 1:**
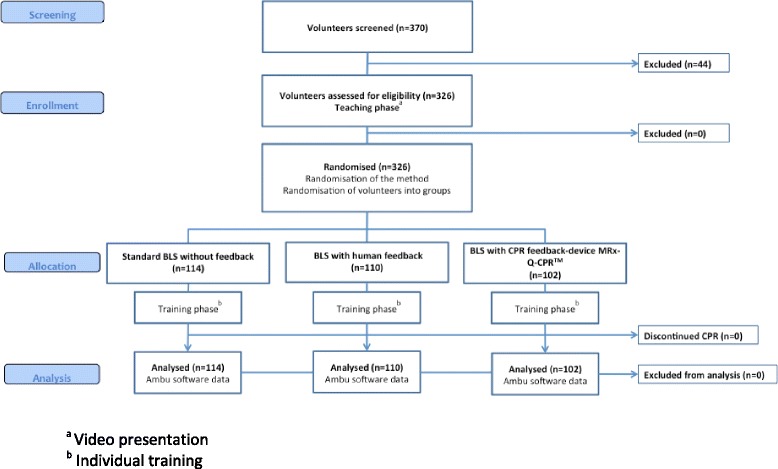
Consort participant flow chart. ^a^ Video presentation. ^b^ Individual training

Out of a total of 370 screened students, we enrolled and randomised 326 participants, no dropouts occurred after randomisation (Fig. 1). Baseline characteristics are shown in Table 1. No significant differences in anthropological parameters, prior resuscitation training or real life CPR experience between the groups was found. Total training time differed between the groups and was shortest for the sCPR group compared to mfCPR (357 vs. 378 s, *p* = 0.010) or hfCPR (357 vs. 572 s, *p* < 0.001). The training video in the hfCPR group was longest, leading also to significantly more time to feel competent with the method compared to mfCPR or sCPR (258 s in hfCPR vs. 210 s in mfCPR, *p* = 0.038; and hfCPR vs. 210 s in sCPR, *p* < 0.001). Differences in sensitivity between the two manikins were negligible and clinically not relevant. No participant had to discontinue the test.

**Table 1:** Baseline characteristics

	Standard BLS (sCPR) (*n* = 114)	QCPR (mfCPR) (*n* = 102)	Human feedback (hfCPR) (*n* = 110)	*P*-value (ANOVA/Chi^2^)
Sex (female), *n*/%	45/40	45/44	51/46	0.569
BMI^1^	21.88 ± 2.27	22.20 ± 2.84	21.77 ± 2.57	0.460
Age [years]	22 (21–23)	22 (21–23)	22 (21–23)	0.910
Real patient resuscitation performed, *n*/%	24/21	22/22	21/19 (*n* = 109)	0.909
Months since last resuscitation	30 (16–36)	20 (8–33)	26 (14–36)	0.438
Last BLS course within *n*/%				
Never	5/4	4/4	1/1	0.267
<6 months ago	28/25	23/23	36/33	0.201
6–12 months ago	13/11	10/10	18/16	0.318
12–24 months ago	43/38	44/43	39/36	0.501
>24 months ago	24/21	20/20	16/15	0.423
Missing	1/1	1/1	0/0	0.596
Duration of training until competent [s]	210 (157–259)^#^	210 (185–320)^†^	258 (200–360)^#,†^	**<0.001**
Total training time [min:s]	5:57 (5:04–6:46)^#, *^	6:18 (5:53–8:08) ^**#, +**^	9:32 (8:34–11:14)^+, *^	**<0.001**

#*p*<0.001 for comparison of sCPR vs. hfCPR

†*p*=0.038 for comparison of mfCPR vs. hfCPR

**#** *p*=0.010 for comparison of sCPR vs. mfCPR

+*p*<0.001 for comparison of mfCPR vs. hfCPR

*p<0.001 for comparison of sCPR vs. hfCPR

Fonts in bold indicate statistical significance (*p*<0.05)

### Compression parameters

ECR was higher in the mfCPR group compared to sCPR (0.35 vs. 0.27, *p* = 0.018), however, not significantly different to hfCPR (0.35 vs. 0.33, *p* = 0.435). Mean compression depth and compression rate were within the recommended range in all three groups. Numerically, the use of the QCPR device resulted in the lowest compression depth of 55 mm and a compression rate of 102 min^−1^. However, the mfCPR group showed the highest percentage of compressions within the recommended range of depth and a tighter control of CPR quality for this parameter through the device.

The percentage of incorrect decompressions was highest in the mfCPR group (33 % in mfCPR vs. 16 % in sCPR, *p* = 0.023; and mfCPR vs. 26 % in hfCPR, *p* = 0.044). The percentage of incorrect pressure points and the occurrence of injuries (blisters on the participants’ palms) were comparable between the three groups. A more detailed description of compression parameters is displayed in Table 2.

**Table 2:** Compression parameters, time related parameters and ventilation parameters

Variables	Standard BLS (sCPR) (*n* = 114)	QCPR (mfCPR) (*n* = 102)	Human feedback (hfCPR) (*n* = 110)	*P*-value sCPR vs QmfCPR	*P*-value sCPR vs hfCPR	*P*-value mfCPR vs hfCPR
Chest compression parameters						
Effective compression ratio (ECR) ^a^	0.27 (0.07–0.36)	0.35 (0.21–0.45)	0.33 (0.10–0.46)	**0.018**	0.078	0.435
Effective compressions [%] ^b^	33 (9–44)	43 (24–55)	41 (13–57)	**0.023**	0.066	0.656
Compression rate [min-1]	105 ± 9	102 ± 5	109 ± 7	**0.004**	**0.008**	**<0.001**
Compression depth [mm]	57 ± 4	55 ± 3	58 ± 3	**0.012**	0.384	**<0.001**
Incorrect decompressions [%]	16 (4–45)	33 (15–47)	26 (2–43)	**0.023**	0.905	**0.044**
Incorrect pressure point [%]	0 (0–5)	0 (0–8)	0 (0–3)		ns*	
Injuries *n*/% ^c^	3/3	1/1	5/5		ns*	
Time related parameters						
Flow time fraction %	81 ± 3	82 ± 2	80 ± 4	**0.003**	0.408	**0.001**
Flow time/min [s] ^d^	49 + 2	49 + 1	48 + 2	**0.003**	0.408	**0.001**
Absolute hands-off time [s] ^e^	61 ± 17	60 ± 15	67 ± 17	0.731	0.053	**0.021**
Time till first chest compression [s] ^f^	3 (2–4)	8 (5–11)	2 (1–3)	**<0.001**	0.172	**<0.001**
Ventilation parameters						
Minute volume [ml/min]	1478 ± 707	1305 ± 655	1276 ± 632		ns*	
Ventilation volume [ml]	260 ± 96	240 ± 95	231 ± 92		ns*	
Ventilation time/min [s]	4 ± 2	3 ± 2	4 ± 2	**0.022**	0.097	0.517
Gastric Inflations, *n*	0 (0–0)	0 (0–0)	0 (0–0)		ns*	

Normally distributed data are presented as mean ± standard deviation; data not following normal distribution are presented as median (IQR). Pairwise comparisons between groups were calculated for significant *p*-values by the means of ANOVA

*P-value <0.05 for ANOVA comparison between all groups; fonts in bold indicate statistical significance (*p*<0.05)

^a^Effective compression ratio was defined as effective compressions [%] multiplied by flow time [%]

^b^Effective compressions were defined as compressions with correct depth (50–60 mm), correct hand position and complete decompression

^c^Observed injuries were blisters

^d^Flow time was defined as the sum of all periods during which chest compressions were performed

^e^Absolute hands-off time was defined as the sum of all periods without chest compressions or ventilation

^f^Time till first chest compression was defined as time from device activation to first chest compression in the mfCPR group and time from beginning of measurement to first chest compression in the sCPR group and the hfCPR group

### Time related parameters

mfCPR was superior regarding flow time fraction compared to sCPR and hfCPR (82 % in mfCPR vs. 81 % in sCPR, *p* = 0.003; and mfCPR vs. 80 % in hfCPR, *p* = 0.001). No significant difference could be found between the sCPR and hfCPR group (*p* = 0.408). Absolute hands-off time was highest in the hfCPR group with significant difference to mfCPR (67 vs. 60 s, *p* = 0.021), whereas hands-off time in the hfCPR group was comparable to the sCPR group (Table 2).

### Ventilation parameters

Minute volume, ventilation volume and gastric inflations were similar among all three groups (Table 2).

### Subjective assessment

In a real-life resuscitation scenario, the majority of participants would prefer CPR with human feedback (42 %), followed by mechanical feedback (27 %). Only 5 % favour CPR without feedback at all, indicating rescuers’ desire for objective evaluation in this setting. At the same time, the participants using the QCPR prompt device rated the difficulty of performing CPR on a scale between 0 and 10 as highest compared to the other groups (8.0 in mfCPR vs. 7.2 in hfCPR, *p* < 0.001; and mfCPR vs. 6.9 in sCPR, *p* < 0.001), while perception of human feedback difficulty did not significantly differ compared to standard BLS (7.2 in hfCPR vs. 6.9 in sCPR, *p* = 0.328).

## Discussion

To the best of our knowledge, this study is one of the few studies to investigate the effectiveness of standardised verbal feedback by trained humans compared to feedback prompt devices and the first study using a two-rescuer BLS scenario. The results demonstrate an equivalent effect of human feedback compared to audio-visual feedback by a feedback prompt device assessed by the compound parameter ECR reflecting CPR quality.

### Feedback prompt devices in clinical practice

The use of audio-visual feedback devices resulted in rescuers providing CPR closer to the ERC recommendations both in manikin and human studies [12]. In accordance with these results, we found a higher CPR quality in the group using the audio-visual feedback device compared to sCPR. Additionally, ECR using the QCPR feedback device was comparable with previous studies [21–23]. Our results extend previous knowledge, since this is the first study testing the HeartStart MRx with Q-CPR-Technology™ in a two-rescuer BLS scenario. The better CPR quality found in this study compared to our earlier studies, where the QCPR technology was used in single-rescuer BLS [19], might be triggered by the fact that additional visual and verbal feedback were provided from the HeartStart MRx monitor. Interestingly, more incorrect decompressions occurred in the feedback prompt device group, which might have been caused by an underestimation of compression depth by the device.

An important issue in the use of CPR devices is the time needed to install the device. In our study the time until the first chest compression was significantly less in the hfCPR group. On the other hand, absolute hands-off time resulting in a significantly smaller flow time fraction was significantly higher in the hfCPR group. The good performance of the feedback prompt device regarding hands-off time and flow time fraction might be attributed to the better visualisation and indication of timing for rescuer change.

### Human feedback

We are only aware of three studies comparing human feedback to feedback through technical measures. In the first study, verbal feedback from an instructor was compared to a “voice advisory manikin” for CPR training of medical students. For overall CPR performance both methods were comparable, however instructor facilitated training was superior for teaching ventilations [15]. As an important limitation, prompts given by a manikin may not be translated into real life scenarios, where patients are unable to give feedback. The second study addressed the effect of verbal feedback on ventilation training in neonatal resuscitation [24], with no overall difference between verbal feedback and a tidal volume monitor. The third study investigated ventilation training in lay BLS courses, reporting that verbal feedback increased hyperventilation and excessive stomach inflation of manikins [25].

Feedback prompt devices provide precise information about the device parameters. Our study showed that human feedback identifies and corrects deviations from ideal CPR parameters in a similar fashion to CPR feedback prompt devices. Experienced rescuers are able to provide a semi-quantitative assessment of the technical skills, such as chest compression depth and rate or sufficiency of decompressions, and quickly recognise incorrect hand position. Although currently there is no evidence supporting the possibility that integrated non-technical skills in human feedback could also improve CPR quality in terms of the five parameters or beyond, it may be assumed that the ability to combine complex information from well-trained human observers in a CPR setting may be beneficial and would lead to an enhanced CPR environment (e.g. recognition of rescuer fatigue and organising the alternation of rescuers providing chest compressions or psychological factors, such as motivation and shared decision making). On the other hand, human feedback would need at least two experienced rescuers.

This raises the question to what extent human feedback can contribute to the improvement of CPR performance in the absence of a feedback prompt device. We found a comparable ECR (quality of CPR) between the hfCPR and the mfCPR group, but on the other hand, no statistical difference between the sCPR and the hfCPR group could be shown. Could more intensive human feedback training improve the performance even more? Or would the combined approach of a feedback prompt device together with feedback-trained humans be the ‘silver bullet’ for the best CPR performance? All in all, we need to start thinking about the interaction of feedback prompt devices and human feedback providers. In summary, human feedback has demonstrated having at least a similar ability to potentially improve CPR quality compared to feedback prompt devices, which are considerably more expensive.

### Limitations and strengths of the study

Naturally, manikin studies simulate cardiac arrest conditions only to a limited extent. On the other hand, simulation allows standardisation of training and testing conditions leading to an enhanced interpretability of performance and CPR quality.

Due to the nature of a single centre study, the generalisation of the results is limited. The study population consisted of recently trained BLS providers with limited experience in real-life BLS. Training time varied inevitably between the groups because of the different teaching content according to the CPR method used (training of verbal feedback was substantially more time-intensive than becoming familiar with the feedback prompt device) and that could definitely be a confounder. Furthermore, the subjective assessment form was not pre-validated.

We did not formally assess the costs of the training. However, future investigations might look at the most suitable duration of training with respect to the best CPR performance after verbal feedback and its cost efficiency compared to commercially available feedback prompt devices requiring large investments.

## Conclusion

This study demonstrated a significant advantage of continuous feedback compared to no feedback during two-rescuer BLS. In addition, verbal human feedback (acquired in a short training session) was not inferior to a mechanical audio-visual feedback prompt device.

## Abbreviations

AED: automated external defibrillator
BLS: basic life support
CPR: cardiopulmonary resuscitation
CR: compression rate
EC: effective compression
ECR: effective compression ratio
FTF: flow-time fraction
hfCPR: human feedback group
ILCOR: international liaison committee on resuscitation
IQR: interquartile range
SCA: sudden cardiac arrest
sCPR: standard BLS group
SD: standard deviation
